# Supplementary material for: An International Study of Correlates of Women’s Positive Body Image
Source: Eur J Investig Health Psychol Educ. 2022 Oct 19;12(10):1521–34. doi: 10.3390/ejihpe12100107 (PMC9601667; doi:10.3390/ejihpe12100107)
Supplement: Supplementary file 1 [file ejihpe-12-00107-s001.zip › ejihpe-1831049-supplementary.pdf]

Supplementary Material

Structural Analysis of the Composite Scales

Confirmatory factor analysis (CFA) was conducted using AMOS 20 version to examine whether the proposed one-factor structure of the BAS-2 adequately represented our data on the total sample. The fit indicators for the default model showed mediocre data-model fit according to Hu and Bentler's (1999) criteria. The model fit indices were: χ^2 = 683.3, χ^2/df = 19.52, comparative fit index (CFI) = .964, normed fit index (NFI)= .962, and root mean square error of approximation (RMSEA) = .084, 90% CI [.078, .089]. To improve the data model fit, we proposed three inter-item errors' covariances (Figure 1). The fit indicators for the modified model showed good data-model fit. The model fit indices were: χ^2 = 381.2, χ^2/df = 11.91, CFI = .980, NFI = .979, and RMSEA = .064, 90% CI [.058, .070]. This modified model was used for further multigroup comparisons.

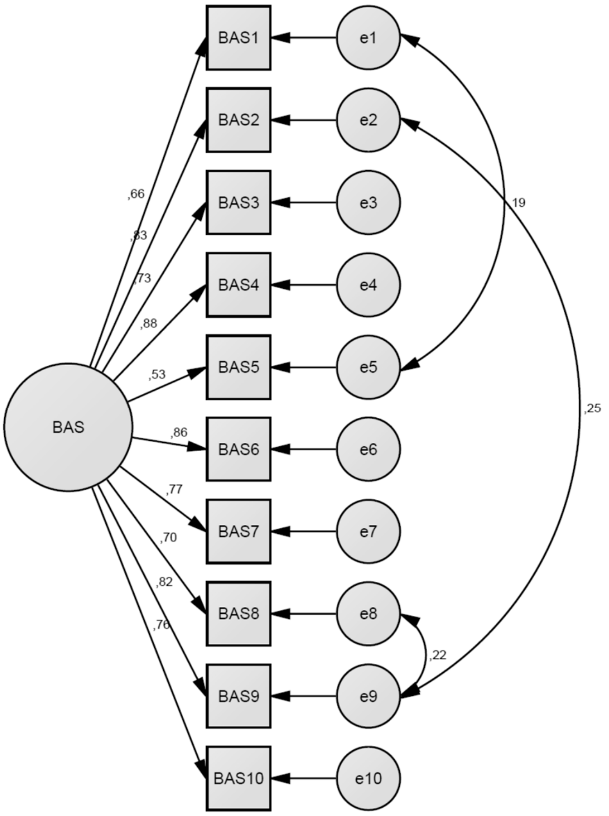

Supplemental Figure S1. Structural model of the BAS-2 with standardized estimates obtained in the complete sample.

To assess the measurement invariance of the BAS-2 model in different countries' subsamples, Multi-Group Confirmatory Factor Analysis (MGCFA) was performed. We tested the typical sequence of nested and hierarchically ordered models by adding parameter restraints one at a time (Cheung & Rensvold, 2002; Vandenberg & Lance, 2000). If two nested models showed a decrease in CFI greater than or equal to .01 or an increase in RMSEA greater than or equal to .01, the more restrictive model should be rejected (Chen, 2007; Cheung & Rensvold, 2002). According to the MGCFA results (Supplemental Table 1), the configural and metric invariance were met. Scalar invariance was met by the ΔRMSEA criteria (.01) but not by the ΔCFI criteria (.03), identifying a weak scalar invariance. Considering that scalar invariance is required to compare latent means (scale scores) across groups, mean differences in BAS scores across countries were interpreted with consideration of possible differences in the meaning of the latent construct across different countries.

Supplemental Table S1

Measurement Invariance of the BAS-2: Model Fit Indices for the Multigroup Models – Country Invariance

Model/ constraints	χ^2	*df*	χ^2/df	$\Delta\chi^2$	Δdf	CFI	NFI	ΔCFI	RMSEA	ΔRMSEA
Unconstrained	688.0	256	2.68			.979	.962		.025	
Measurement weights	816.3	319	2.56	128.3*	63	.972	.955	–.007	.024	–.001
Measurement intercepts	1581.8	388	4.08	765.6*	69	.933	.913	–.042	.034	.010
Structural covariance	1607.2	395	4.07	25.3*	7	.932	.911	–.002	.034	.000

Note. CFI = comparative fit index; NFI = normed fit index; RMSEA = root mean square error of approximation. *$p < .01$.

CFA was also conducted to check the proposed one-factor structures of the Self-Reported Study Performance (SRSP) scale, Educational Aspiration scale (CAS-R/Ed), and Rosenberg Self-Esteem Scale (RSES). These analyses were performed only on the total

sample of the participants, as we were interested in correlational and mediational effects, not in the countries' means comparisons. The fit indicators for the default one-factor models showed average data-model fit according to Hu and Bentler's (1999) criteria. To improve the data model fit, we proposed three inter-item errors' covariances for the SRSP and CAS-R/Ed scales and four inter-item errors' covariances for the RSES. The fit indicators for the modified models showed good data-model fit for all three scales (Supplemental Table 2). These findings enabled the use of the scales' total scores in correlational analyses of the total sample.

Supplemental Table S2

CFA Results for the Self-Reported Study Performance Scale, Educational Aspiration Scale, and Rosenberg Self-Esteem Scale on the Total Sample

Model/ constraints	*N*	χ^2	*df*	χ^2/df	CFI	NFI	RMSEA (90% CI)
SRSP	2634	73.8	6	12.3	.992	.991	.065 (.053–.079)
CAS-R/Ed	2616	247.5	17	14.5	.978	.977	.072 (.064–.080)
RSES	2638	514.0	31	16.6	.959	.956	.077 (.071–.082)

Note. SRSP = Self-Reported Study Performance Scale; CAS-R/Ed = Educational Aspiration subscale of the Career Aspiration Scale-Revised; RSES = Rosenberg Self-Esteem Scale; CFI = comparative fit index; NFI = normed fit index; RMSEA = root mean square error of approximation; CI = confidence interval.

References

Chen, F. (2007). Sensitivity of goodness of fit indexes to lack of measurement invariance. *Structural Equation Modelling*, *14*(3), 464–504. https://doi.org/10.1080/10705510701301834

Cheung, G. W. & Rensvold, R. B. (2002). Evaluating goodness-of-fit indexes for testing measurement invariance. *Structural Equation Modeling*, *9*, 233–255. https://doi.org/10.1207/S15328007SEM0902_5

Hu, L. & Bentler, P. M. (1999). Cutoff criteria for fit indexes in covariance structure analysis: Conventional criteria versus new alternatives. *Structural Equation Modeling: A Multidisciplinary Journal, 6*(1), 1-55. https://doi.org/10.1080/10705519909540118

Vanderberg, R. J. & Lance, C. E. (2000). A review and synthesis of the measurement invariance literature: Suggestions, practices, and recommendations for organizational research. *Organizational Research Methods*, *3*, 4–70. doi:10.1177/109442810031002
